# Analysis of Ni-Cu Interaction in Aluminum-Based Alloys: Hardness, Tensile and Precipitation Behavior

**DOI:** 10.3390/ma17184676

**Published:** 2024-09-23

**Authors:** Ehab Samuel, Agnes M. Samuel, Victor Songmene, Herbert W. Doty, Fawzy H. Samuel

**Affiliations:** 1 Département des Sciences Appliquées, Université du Québec à Chicoutimi, Saguenay, QC G7H 2B1, Canada; ehabfhsamuel@gmail.com (E.S.); agnesmsamuel@gmail.com (A.M.S.); 2 Département de Génie Mécanique, École de Technologie Supérieure, Montreal, QC H3C 1K3, Canada; victor.songmene@etsmtl.ca; 3 Materials Technology, General Motors Global Technology Center, Warren, MI 48092, USA; herb.doty@gm.com

**Keywords:** Al–Si alloys, Ni, Zr, HR-TEM, FESEM

## Abstract

The present work was aimed at quantifying the effects of Ni addition in the range of 0–4% together with 0.3%Zr on the hardness and the tensile properties, volume fraction of intermetallics, and changes in size and distribution of phase precipitation in Sr-modified Al-9%Si-2%Cu-0.6%Mg cast alloys. The study was mainly carried out using high-resolution FESEM and TEM microscopes equipped with EDS facilities. Samples were solidified at the rate of ~3 °C/s and examined at different aging conditions. The investigations are supported by thermal analysis carried out at a solidification rate of ~0.8 °C/s. The results revealed that the main compositions of the Ni-based phases are close to Al_3_(Ni,Cu), Al_3_CuNi, and Al_3_Ni. An Al_3_Ni_2_Cu_2_ phase was also detected in the 4%Ni alloy. The Cu–Ni phases were observed to precipitate, covering the surfaces of pre-existing primary Al_3_Zr particles. The TEM analysis indicated the magnitude of the reduction in both size and density of the precipitated Al_2_Cu phase particles as the Ni content reached 4%, coupled with a delay in the transition from coherent to incoherency of the Al_2_Cu precipitates.

## 1. Introduction

In general, the mechanism of alloy hardening by precipitation results in the formation of coherent clusters of solute atoms; that is, the solute atoms gathered in a cluster must always have the same crystal structure as the phase of the solvent. This then creates a lot of distortion because the crystal parameters of the precipitates can be slightly different from those of the metal matrix [1,2,3,4,5,6,7,8,9]. The Guinier–Preston or GP zones (solute clusters) and coherent precipitates are effective in blocking dislocations from moving, given the significant stress and strain fields that surround them. The dislocations, to pass the coherent precipitates, have no choice and must shear them, hence increasing the stress required to deform the part [10,11,12,13,14,15,16,17,18,19].

Addition of copper (Cu) to aluminum-based alloys would lead to precipitation of Al_2_Cu phase particles during the solidification process. Dissolution of Al_2_Cu fine particles during solutionizing treatment may occur in 2 h at 495 °C solutionizing temperature. Which is not the case for massive Al_2_Cu particles where segregation may take place, leading to incipient melting and hence poor mechanical properties. The combined addition of Cu and Mg will result in balancing the alloy mechanical strength through the mutual precipitation of Al_2_Cu and Mg_2_Si phase particles [20,21,22,23,24].

Zirconium (Zr) may be used in Al alloys to refine the grain structure due to the precipitation of fine coherent dispersoids (i.e., Al_3_Zr), which obstruct dislocation motion, leading to enhancement of the high temperature mechanical properties of aluminum alloys [25]. In order to increase the volume fraction of Al_3_Zr precipitates, and based on the phase diagram of Al–Zr, the concentration of Zr in the alloys investigated in this study was kept at around 0.3 wt% [26]. According to Nakamura et al. [27], the addition of Ag to Al-based alloys containing Mg reduces the size of the precipitates and promotes the precipitation of numerous fine-scale AlMgAg particles, leading to improvement of the alloy mechanical properties.

Nickel (Ni) has a relatively lower contribution to the alloy mechanical performance of aluminum–silicon (Al–Si)-based alloys compared to that produced by the addition of copper (Cu) and magnesium (Mg). Nickel has traditionally been used in a proportion of 2–3% because it was believed to improve the mechanical properties of alloys used at high temperatures [26,27,28,29]. The nickel compounds dispersed inside the microstructure suggest that the mechanical properties are improved by the presence of nickel [30,31,32,33]. However, the inter-particle spacing of Al_3_Ni compounds being considerable, of the order of 50 µm, this large distance would not allow perceptible dispersion hardening. Furthermore, it could be possible that the nickel compounds are formed via a fiber hardening process, but this would not make a significant contribution since the network of silicon fibers in large volume would annihilate this effect of nickel [34,35,36,37].

The use of Ni is limited to alloys used in atomic reactors due to its high neutron absorption, but in other applications, its addition is desirable: combined with Fe, for example, it improves resistance to high vapor pressure corrosion [38]. It was reported that the addition of Cu (1.0 to 1.8%) and Ni (1.0 to 1.6%), or just Cu, to eutectic Al–Si alloys produces multicomponent phases of the following types: Al_40_Si_7_(Fe,Mn)_11_Cu, Al_40_Si4Fe_2_MnCuNi_11_, and Al_40_Si(Fe,Mn,Cu,Ni)_13_ [39,40,41,42].

This present study is in continuity with the previous work carried out by the current authors [43,44,45,46] in order to explore in depth the role of the interactions between the alloying elements in determining the final phases in the microstructure of the Al–Si–Cu–Mg alloys [47] and the extent to which they affect the alloy strength. Based on these studies, zirconium (Zr) will also be added to the base alloy containing 9%Si-2%Cu, 0.6%Mg, 0.15%Fe, followed by Ni addition, to study the combined effect of Zr on the strength properties of the 354 alloy at ambient and high temperatures. Transmission electron microscopic analysis will be used to monitor the changes in the size, density, and distribution of the precipitates formed and their crystallographic characteristics.

## 2. Experimental Procedure

The Al–Si–Cu–Mg base alloy was received in the form of ingots (13 kg each). Alloying elements were added using Al-25%Ni, Al-20%Zr, Al-11%Sr, and Al-5%Ti-1%B master alloys, as well as pure silver (Ag), to the molten commercial base alloy to produce the four compositions listed in Table 1. Before casting, the melt temperature was ~750 ± 5°C. For each alloy prepared, the molten bath was degassed for about 20 min with a graphite impeller turning at a speed of ~130 rpm, applying argon gas. Samplings for chemical composition were carried out prior to casting. The chemical analysis was done using a spectral analysis technique. The chemical analysis was carried out using a Spectrolab-JrCCD Spark Analyzer (Spectro Analytical Instruments inc., Mahwah, NJ, USA). The average chemical composition is three burns per alloy sample. The average chemical compositions (three burns per alloy sample) are reported in Table 1.

**Figure 1:**
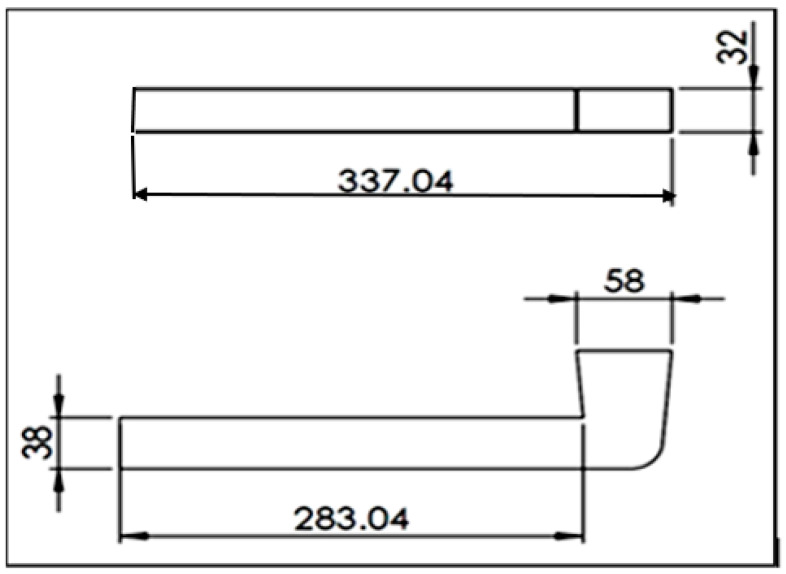
L-shape casting mold (all dimensions are in mm; solidification rate was ~3 °C/s).

Thereafter, the melt was cast into a preheated (450 °C) metallic mold—Figure 1. This mold is characterized by a large liquid metal entry (riser) that will accommodate the shrinkage cavity, resulting in a sound, uniform long bar with a square cross section. Another set of castings was prepared using an ASTM B-118 type permanent mold (gage length coated with a thin layer of ultra-fine boron-nitride). The mold was preheated at 450 °C, and the solidification rate was estimated at 7 °C/s. All prepared samples were heat treated using an air circulation electrical oven. Details of the applied heat treatments will be mentioned further on in the text.

The heat treatment was carried out using a Lindberg Blue M electric furnace (Therno Fisher Scientific, Waltham, MA, USA). It is important to mention that the time elapse between removal of the test-bar bundles from the furnace and quenching was at most ~5 s. Tensile bars were solutionized at 511 °C for 8 h, followed by quenching in warm water (60 °C). Table 2a,b summarizes the applied working conditions.

Following heat treatment, all tensile test bars were pulled to fracture at ambient temperature (and an MTS servo-hydraulic mechanical testing machine) at a strain rate of 4 × 11^−4^ s^−1^. An attachable extensometer (strain gauge) was used to measure the deformation that took place in the samples during deformation, where the data acquisition system attached to the machine converts it to an accurate measure of the percentage elongation. The data acquisition system provides the tensile properties in terms of ultimate tensile strength (UTS), yield strength (YS), and the percentage elongation to fracture (%El). Five test bars for each alloy/condition were tested, and the average values of ultimate tensile strength (UTS), 0.2% offset yield strength (YS), and percentage elongation to fracture (%El) were reported as representing the tensile properties of the corresponding alloy/condition. Some of these bars were tested at 240 °C [48].

A high-sensitivity Type-K (chromel–alumel) thermocouple, which has to be insulated using a double-walled ceramic tube, is attached to the center of the graphite mold. The temperature–time data is collected using a high-speed data acquisition system linked to a computer system to record the temperature-time data every 0.1 s [43]. From this data, the solidification curves and the corresponding first derivative curves for a number of selected alloys were plotted to identify the main reactions occurring during solidification with the corresponding temperatures. The various phases, which constitute the microstructure of each alloy, were expected to be revealed as well. The solidification rate determined from the dendrite arm spacing is about 0.8 °C/s.

Vickers hardness measurements were carried out using an automatic CLEMEX micro-hardness tester (Brossard, Montreal, QC, Canada), and an indentation load of 110 gf was applied for 11 s. Sixteen indentations were made on each specimen to analyze the hardness distribution. Scanning electron microscopic (SEM) analysis was carried out employing a Hitachi SU-8230 FESEM (Chiyoda City, Japan) equipped with a Bruker Quantax Flat Quad EDS detector (Madison, WI, USA) to investigate phase evolution in the heat-treated and reference samples. Transmission electron microscopy (TEM) was used employing a FEI Tecnai G2 F20 electron microscope (Sensitive Instrument Facility, Ames, IA, USA), equipped with an advanced control system that permits the integration of an EDAX™ chemical analysis system, and scanning transmission electron microscopy (STEM). The microscope was operated at an accelerating voltage of 20 kV.

The preparation process of samples for high-resolution transmission electron microscopy includes sectioning a very thin disc of about ~300 µm from the original thick samples that were solidified at a rate of 0.35 °C/s. In order to avoid mechanical deformation of the disc, cutting was carried out using a diamond disk at a low speed. Thereafter, 3-mm diameter discs were prepared from the thinned discs. In order to further thin the prepared foils, an UniMill IV7 ion milling machine (Technoorg Linda Co., Limited, Budapest, Hungary) was employed to produce transparent foils.

The main purpose of applying the DSC analysis system was for identification of the phases that were precipitated during the solidification process at a rate of 0.355 °C/s. The main purpose of using such a low solidification rate was to increase the solidification time and hence the size of the precipitated phase in order to obtain more accurate and reliable data. The thermal analysis method (DTA analysis) was carried out in the same manner the 354 alloy heats were prepared. In this case, the initial materials were melted in a 2-kg SiC crucible by means of an electrical furnace at 750 °C, then poured in a graphite crucible pre-heated at 600 °C. As a result, a solidification rate of about 0.35 °C/s could be achieved.

## 3. Results and Discussion

### 3.1. Microhardness and Tensile Testing

**Figure 2:**
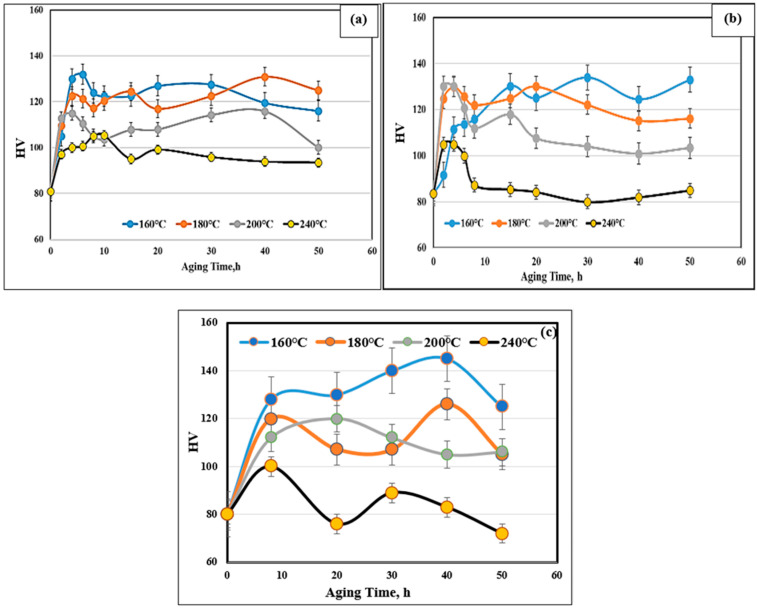
Variation in microhardness of (**a**), B (**b**) BS, (**c**) B2N alloys as a function of aging temperature and time.

Figure 2 shows the effect of Sr and Ni addition on the aging behavior of the 354 alloy in the temperature range of 160–240 °C and for aging times varying from 0 to 50 h, with 0 h representing the T4 heat treatment. The samples were solutionized at 495 °C for 5 h prior to quenching in warm water (60 °C); 0 h corresponds to T4) [48,49,50].

Abdelaziz et al. [48] performed a series of aging treatments on 319 alloy containing 6.8%Si-3%Cu-0.3%Mg. The results revealed that all hardness curves exhibited wavy patterns composed of multiple peaks, whereas the 356 alloy (containing 7%Si-0.35%Mg) revealed only a single peak. Zhou et al. [49] confirmed the observations made on the 319 alloy. Mohamed et al. [50] interpreted the results in terms of the precipitation of Al_2_Cu and Al_2_CuMg phase particles as well as the increased bonding of the Si particles with the matrix, where the thermal energy is sufficient to precipitate such hard phases. The growth of the phase precipitates with an increase in the aging time, which would cause softening. The hardness measurements presented in Figure 2a,b show the clear lack in the start of hardening for the BS alloy at 160 °C.

Joenoes and Gruzleski [51] reported that in Mg-containing alloys (Mg > 1%), it is likely that Sr would react with Mg, forming the Mg_2_SrAl_4_Si_3_ phase, which precipitates prior to the eutectic reaction; this would explain the occurrence of such aging behavior. Another point to emphasize is the fast particle coarsening rate on aging at 240 °C exhibited by the BS alloy compared to that shown by the B alloy, which may be attributed to a lack of free Mg, resulting in a lesser number of strengthening precipitates.

**Figure 3:**
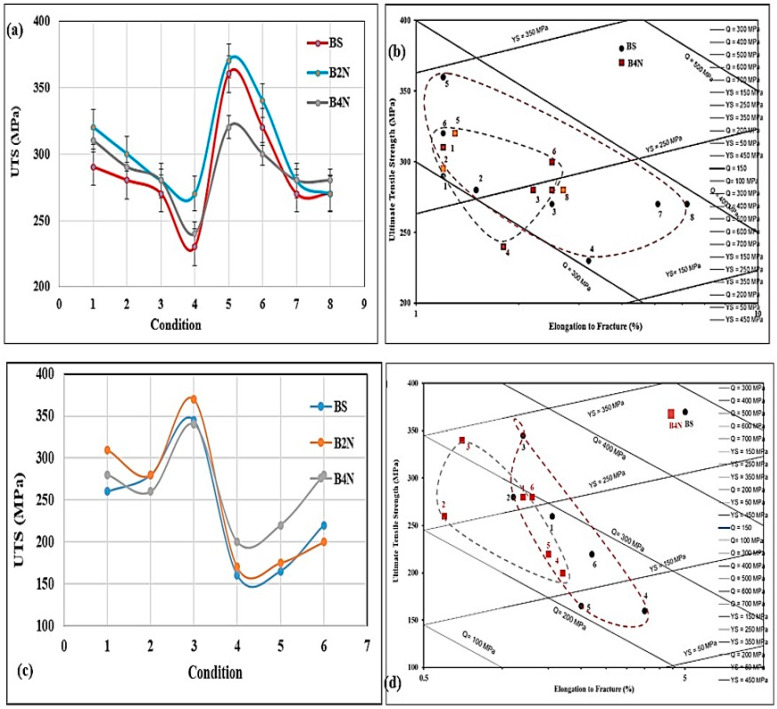
Variation in the alloy tensile properties as a function of the applied heat treatment and testing temperature: (**a**) ultimate tensile strength (Table 2a)—RT, (**b**) Q-chart of data in (**a**), (**c**) ultimate tensile strength (Table 2b)—240 °C, (**d**) Q-chart of data in (**c**). The conditions 1 to 8 and 1 to 6 marked on the X-axis in (**a**,**c**) refer to the heat treatment conditions listed in Table 2. The dashed lines in (**b**,**d**) outline how the quality and strength of the alloy evolves with the heat treatment conditions.

The microstructure shown in Figure 2c for the B2N alloy reveals a large volume fraction of Ni-based phases. Whereas it is known that Cu and Ni can dissolve in each other, producing Cu–Ni compounds that are insoluble in the surrounding Al matrix (marked X). Figure 3a depicts the variation in the ultimate tensile strength (UTS) of the three studied alloys (i.e., BS, B2N, and B4N) following different aging treatments as described in Table 2a. As can be seen, the highest resistance to softening is associated with alloy B4N in the T5- and T7-treated conditions. The lowest values were obtained in the T5- and T7-treated conditions for BS and B2N alloys.

The T7-treated B2N and B4N alloys, with 2 and 4 wt% Ni, respectively, show the best strength values after 200 h aging at 240 °C following solutionizing at 495 °C for 5h. This observation may be interpreted in terms of the simultaneous precipitation of Al–Cu–Ni and Al_3_Ni phases in all Ni-containing alloys (B2N, B4N). In particular, alloy B4N reveals the best resistance to softening and highest strength values, likely due to the uniform distribution of (Al-Al_3_Ni) eutectic structure. These observations are better represented by the quality index chart following the concept proposed by Drouzy et al. [52] and Jacob [53], as shown in Figure 3b for the BS and B4N alloys. It is evident from Figure 3b that the data for the BS alloy, expands over an area almost twice that of the B4N alloy, where all spots are seen to fall in clusters. However, at prolonged aging at 240 °C, alloy B4N offers better resistance to softening compared to that produced by the BS alloy.

Table 2b lists the heat treatment conditions of alloys tested at 240 °C. From Figure 3c, it may be seen that testing at 240 °C resulted in a noticeable reduction in both YS and UTS strength parameters compared to the values obtained at room temperature. On the other hand, as Figure 3b reveals, increased values of ductility were obtained when testing at 240 °C, compared to those reported from ambient temperature tests. These observations may be attributed to the softening associated with tensile testing at high temperatures, consequent to coarsening and subsequent reduction in density of the main strengthening precipitates, e.g., Al_2_Cu, Mg_2_Si, and Al_2_CuMg.

One of the main advantages of Ni addition is the improvement in alloy resistance to softening at high-temperatures, i.e., 240 °C [54]. In the present work, alloy B4N exhibited the highest resistance to softening during high temperature testing. In the T7-treated condition, alloy B4N (containing 4 wt% Ni) exhibited a reduction in YS by about 65 MPa when tested at 240 °C. In comparison, alloy B2N (with 2 wt% Ni) displayed the lowest resistance to softening, with a loss in YS of about 140 MPa. The high resistance to softening at 240 °C shown by alloy B4N may be attributed to precipitation of fine Al_3_Ni phase (Al-Al_3_Ni) with the increase in Ni-content from 2 to 4 wt% Ni. It is well documented that a finely distributed Al_3_Ni phase, e.g., as that observed in alloy B4N, would enhance the elevated temperature strength significantly compared to its coarsened size observed in the microstructures of the 2 wt% Ni-containing B2N alloy [55,56].

The quality index values of the BS and B4N alloys are depicted in Figure 3d following the concept of Drouzy et al. [52] for selected heat treatment conditions as described in Table 2. Both alloys revealed a marked drop in Q-values at 240 °C due to a major reduction in the corresponding UTS levels despite their improved ductility values. The Q values of alloy B4N are low, in general, except for the Q value for the T7-treated condition when tested at 240 °C due to the noticeably increased strength of alloy B4N at this temperature. Table 3 lists the Reactions 1 through 8 and the corresponding phases and reaction temperature ranges observed for the base alloy B (or BS) and B2N based on ref. [57].

**Figure 4:**
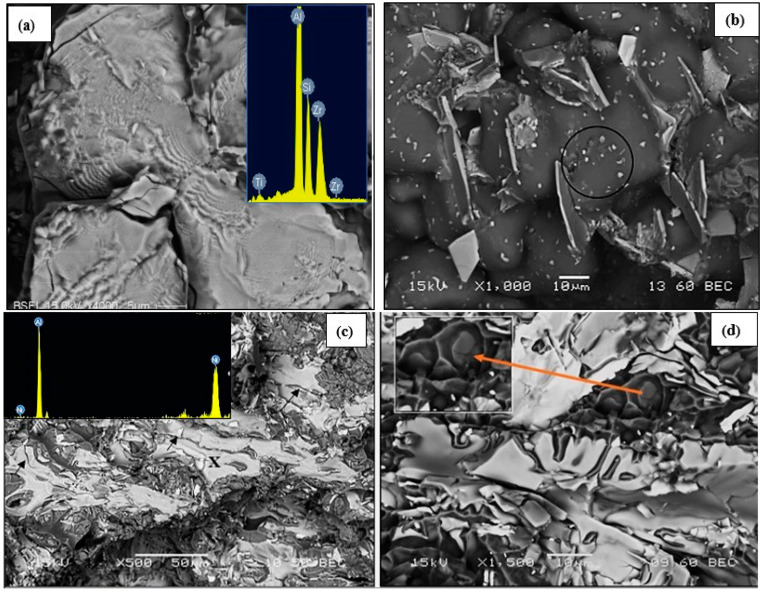

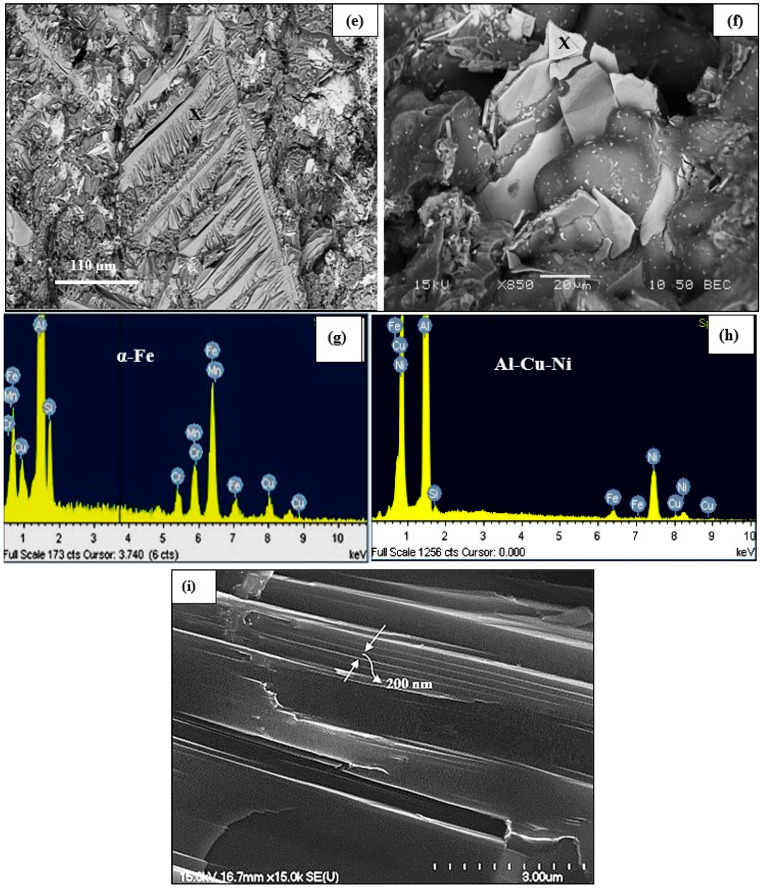
Fracture surface of BS alloy in the T7 condition, tested at: (**a**) 25 °C, (**b**) 240 °C; fracture surface of the B4N alloy in the T7 condition, tested at: (**c**) 25 °C, (**d**) 240 °C—note the precipitation of Ni-rich phase particles in the form of dendritic cells; (**e**,**f**) precipitation of (**e**) α-Fe and of Al_2_Cu phase particles; (**g**,**h**) EDS spectra corresponding to areas marked X in (**e**,**f**), respectively; (**i**) stacking faults.

The fractured surface of T7-treated alloy BS after testing at room temperature, seen in Figure 4a, displays a fine dimple structure together with bright Al_x_(Zr, Ti)Si precipitates. The inset shows the EDX of these precipitates; some of them appear in star-like form. The high temperature-tested sample of the alloy shows a cracked Alx(Zr,Ti)Si particle (arrowed) in the high magnification image displayed in Figure 4b. Several fine precipitates of mostly Al_2_Cu particles are also observed, bordering the block-like Alx(Zr,Ti)Si particle in the figure.

Figure 4c,d display the fracture characteristics of the T7-heat-treated alloy B4N when tested, respectively, at ambient and high temperatures. Large Ni-rich phase particles are observed in both cases. The EDS spectrum taken from the spot X in Figure 4c shown in the inset indicates that these are Al_3_Ni particles. The black arrows in the figure point to their fractured nature, while the brittle nature of the alloy is evidenced by the finely dimpled network surrounding the Al_3_Ni particles. As Table 7 indicates, while high temperature testing hardly increased the volume fraction or size of the Ni-based phases (Figure 4d), the size of the dimple structure was noticeably bigger (see inset micrograph). However, any improvement due to the increased aluminum matrix area was offset by the high-volume fraction (about 11.5%—Table 7) of brittle intermetallics present, in addition to precipitation that may have occurred during testing as can be seen at the bottoms of the dimples (inset micrograph—orange arrow).

Consider the atomic numbers of Fe, Ni, and Zr: 26, 28, and 40, respectively. The backscattered electron image presented in Figure 4e reveals the fracture of α-Fe in its dendritic shape similar to that featured in Figure 4d except for its grayer color when compared to the bright appearance of the Ni-based intermetallic phase due to the obvious difference in the atomic numbers of the two phases. The fracture surface depicted in Figure 4f demonstrates two main observations: the presence of the Al–Ni–Cu phase in the form of platelets coupled with a large density of fine Al_2_Cu particles. Since it is a fracture surface, it is difficult to estimate the density of the precipitates. Figure 4g and Figure 4h are the EDS spectra corresonding to Figure 4e and Figure 4f, respectively, obtained from the spots marked X in each case. Occasionally stacking faults were seen in the fracture surface, as displayed in Figure 4i.

### 3.2. Microstructural Characterization of As-Cast Alloys

#### 3.2.1. Thermal Analysis

**Figure 5:**
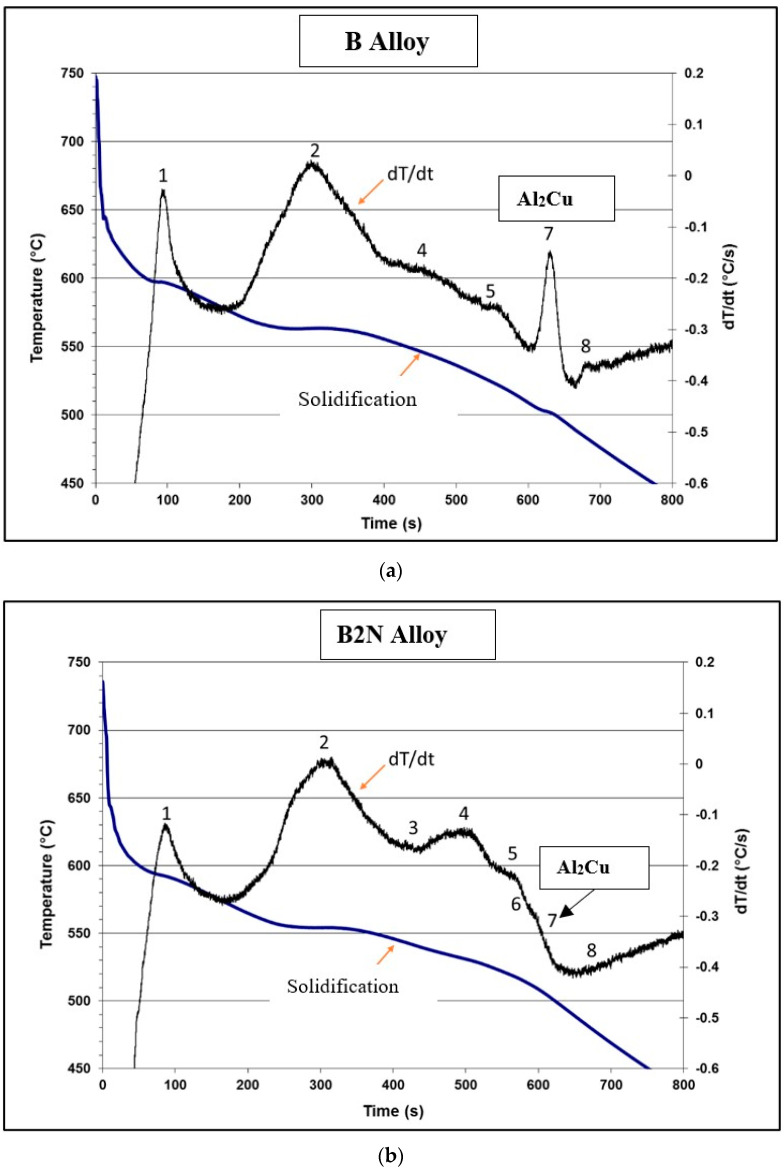

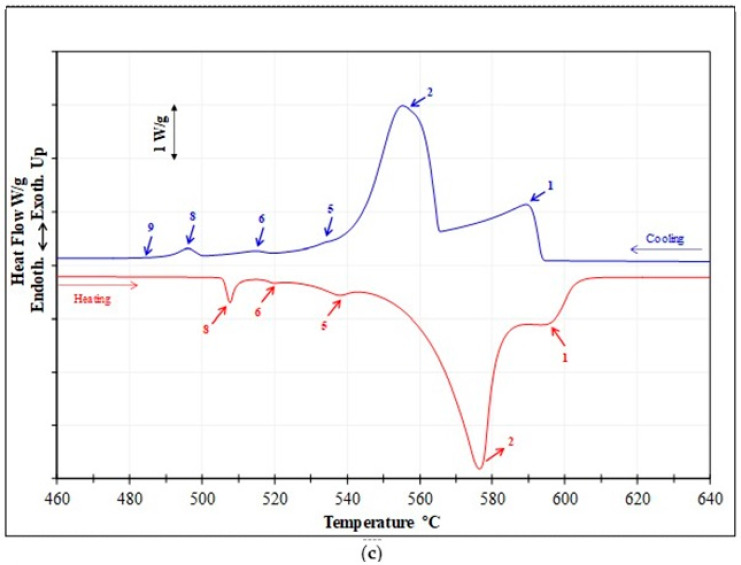
(**a**) Solidification curve (blue) and its first derivative (black) obtained from alloy B. (**b**) Solidification curve (blue) and its first derivative (black) obtained from alloy B2N (containing 2 wt% Ni + 0.25 wt% Zr). (**c**) DSC solidification curves of the BS alloy (see Table 6 for reactions).

The base alloy B is an Al–Si–Cu–Mg alloy, with its composition of 8.4 wt%Si, 0.15 wt%Fe, 2.1 wt%Cu, 0.59 wt%Mg, and 0.15 wt%Ti. It is expected that Cu-rich, Mg-rich, and Fe-rich intermetallic phases will mainly precipitate during solidification. Referring to Table 3, which lists reactions expected in such alloys, and as seen from Figure 5a, solidification commences at 598 °C with the formation of the dendritic Al network (reaction 1), followed by the Al–Si eutectic at 560 °C and post-eutectic B-Al_5_FeSi phase (reaction 2), Mg_2_Si at 540 °C (reaction 4), and the beta-Fe to pi-Fe phase transformation at 525 °C. Finally, precipitation of the Al_2_Cu and Q-Al_5_Mg_8_Cu_2_Si_6_ phases occurs at 498 °C and 488 °C when solidification is completed. The WDS analysis of the reported phases is listed in Table 4.

The solidification curve for alloy B2N containing 2 wt% Ni + 0.25 wt% Zr is shown in Figure 5b. Table 5 presents the chemical compositions of the phases examined in alloy B2N as obtained from the WDS analysis of the sample used. Note the disappearance of reaction #7 (precipitation of Al_2_Cu) in Figure 5b.

DSC solidification and melting curves of the base BS alloy studied are provided in Figure 5c, displaying the suggested endothermic and exothermic transformation reactions. These reactions are listed in Table 6.

**Table 6:** Suggested reactions taking place during solidification/melting of the BS alloy [24,54,57].

Reaction #	Transformation Temperature (°C) [24,54,57]	Reactions Occurring during Solidification and Melting	Temperatures Reported during Solidification (°C)	Temperatures Reported during Melting (°C)
			BS	BS
1	600–597	α-Al dendritic network	590	595
2	560–558	Al-Si eutectic Post-eutectic β-Al_5_FeSi phase α-Al_15_(Fe,Mn)_3_Si_2_ phase for Mn-containing alloys	556	576
3	555–556	Al_9_FeNi phase	-	-
4	546–553	Al_3_Ni phase	-	-
5	540–538	Mg_2_Si phase	534	538
6	525–523	Transformation of β to π-Al_8_Mg_3_FeSi_6_ phase	516	520
7	523–520	Al_3_CuNi phase	-	-
8	500–496	eutectic Al-Al_2_Cu phase	496	507

#### 3.2.2. Optical and SEM Investigations of as Cast Alloys

While the literature reports many studies on the effects of Ni addition to Al–Si alloys [55,56,57,58] in relation to the microstructure/mechanical properties, most of them are focused on Ni-phase precipitates, neglecting other aspects such as grain refining, for example. In the present study, a certain amount of grain refining was observed in the B2N alloy (15–20%), much less than that anticipated with 2% Ni addition. Wangi and Reif [58] attribute this refining to the constitutional undercooling effect of Ni.

**Figure 6:**
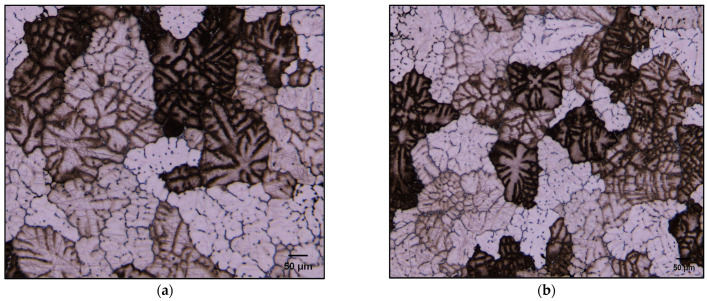
Macrostructure of grains in the as-cast: (**a**) B alloy, (**b**) B2N alloy. Samples were lightly etched to reveal the secondary dendrite arm spacing (SDAS).

Table 7 lists the volume fraction of undissolvable intermetallics in the present alloys in the as-cast condition and after quenching from the solutionizing temperature (T4 tempered condition). Taking into consideration that all alloys (except the B (or BS) base alloy) contain about 150 ppm Sr, Figure 6 compares the changes in the microstructure of BS and B2N alloys in the as-cast condition. It is observed that Ni may lead to some Si particle refinement as seen in the circled areas but not enough to classify Ni as a grain refiner similar to that obtained using approximately 0.1–0.2%Ti in the form of Al_3_Ti [59] or TiB_2_ [60,61,62,63,64,65].

**Figure 7:**
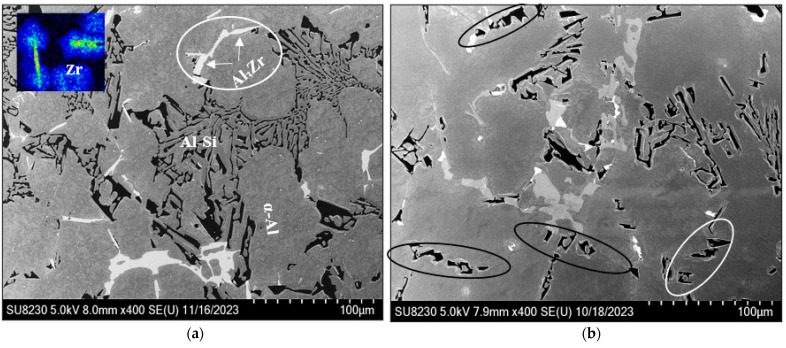

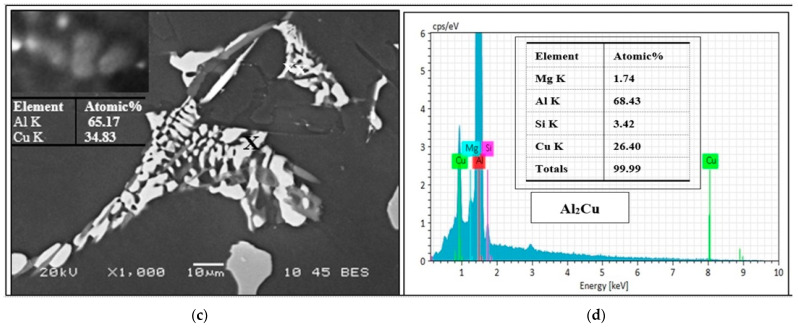
Backscattered electron micrographs of (**a**) BS and (**b**) B2N alloys in the as-cast condition revealing precipitation of Al_3_Zr and eutectic Si phase particles in BS alloy—white circled area in (**a**), and eutectic Si particles in B2N alloy—see the white or black circles in (**b**); (**c**) Backscattered electron micrograph of BS alloy in the as-cast condition; (**d**) EDS spectrum corresponding to the white area in (**c**) marked X.

Garza-Elizondo et al. [66] reported that the as-cast microstructure of 354 alloy consists of dendrite arm spacings (DAS) in the range 50–70 µm using a close-to-equilibrium solidification rate, which was also used in the present work. Figure 7a shows the microstructure of the as-cast BS alloy, revealing the precipitation of a large volume of Al_2_Cu phase particles surrounded by well-modified eutectic Si particles. The associated EDS of the area marked X in Figure 7c reveals a strong peak due to Mg, indicating the possibility of the precipitation of Q-Al_5_Cu_2_Mg_8_Si_6_ phase [67,68,69], as listed in Table 3 and shown in the inset micrograph at the top left in Figure 7a.

Garza-Elizondo and coworkers [66] reported that in the 354 alloy they investigated, the as-cast structure displayed DAS or dendrite arm spacing values of 50–70 um when the alloy was solidified under equilibrium solidification conditions. Such conditions were also adopted in the present study. The as-cast microstructure of the BS alloy displayed in Figure 7a shows a large amount of Al_2_Cu precipitates surrounded by fine Al–Si eutectic regions. The EDS spectrum taken from X in Figure 7c, depicting a concentrated region of Al_2_Cu particles in the alloy, also exhibits a peak due to Mg, which indicates the possible presence of the Q-Al_5_Cu_2_Mg_8_Si_6_ phase, as listed in Table 3 and shown in the inset micrograph at the top left in Figure 7c.

**Figure 8:**
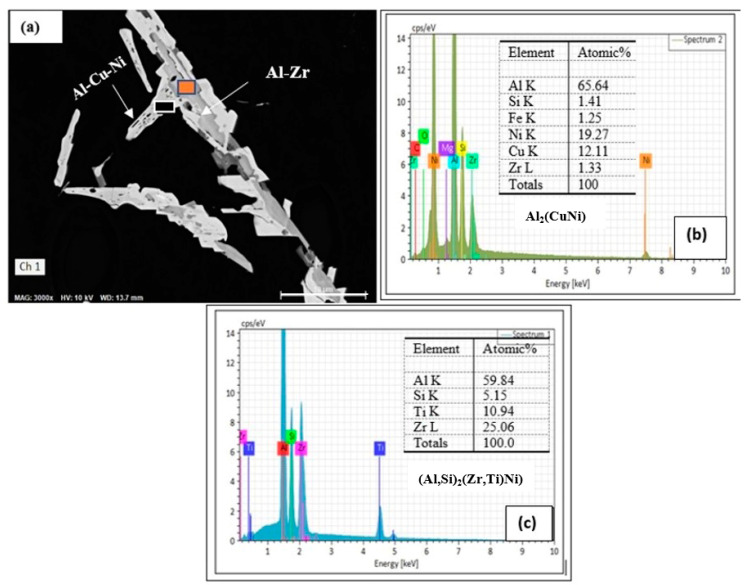
(**a**) Backscattered electron micrograph of the B2N alloy in the as-cast condition, (**b**,**c**) EDS spectra corresponding to the black and orange squares in (**a**), respectively. The energies of the Lα lines of Cu and Ni are very close (0.933 and 0.853 keV, respectively), leading to overlapping of the lines.

It may be inferred from the Al–Zr binary phase diagram that the melting temperature of Al-0.3% Zr is about 750 °C. Therefore, in the present work, the temperature of the melt was raised to 820 °C to allow for the dissolution of Zr. Thereafter, the temperature was lowered to 750 °C before starting the degassing process. During solidification and before reaching the peritectic reaction at 660 °C, primary Al_3_Zr particles would precipitate in the form of dispersoids [70,71,72]. Figure 8a shows that in the B2N alloy, it is possible that Al–Ni–Cu compound may precipitate, covering the Al_3_Zr particles. In addition, Ni would also react with Zr, forming Ni_5_Zr compound [73,74]. On the other hand, Cu would react with both Ni and Zr (Cu_9_Zr_2_) [75,76]. Figure 8b is the EDS spectrum corresponding to the black rectangular area in Figure 8a, revealing peaks of Al, Ti, Cu, Zr, and Ni, whereas the EDS spectrum obtained from the orange square reveals strong Zr peaks, as seen from Figure 8c.

**Figure 9:**
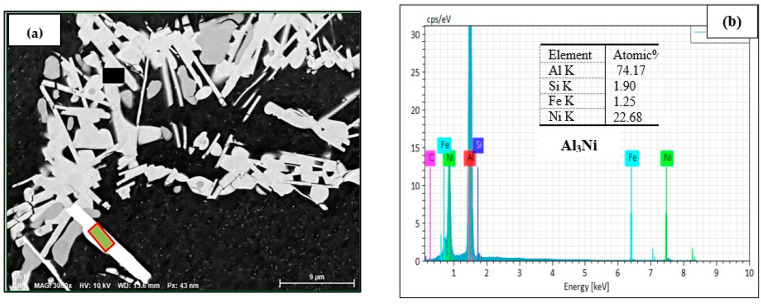

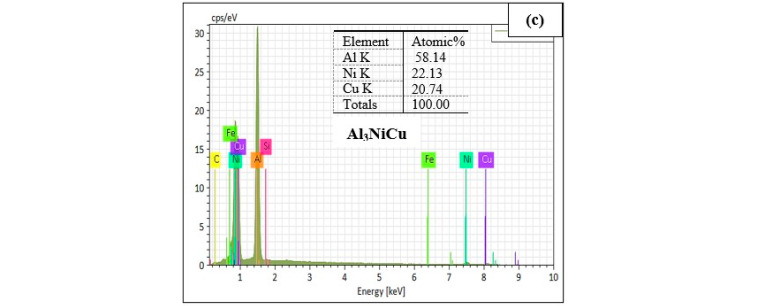
(**a**) Backscattered electron micrograph of the B2N alloy in the as-cast condition; (**b**) EDS spectrum corresponding to the black square in (**a**); (**c**) EDS spectrum corresponding to the rectangle outlined in red in (**a**), showing the presence of Al_3_Ni, Al_3_CuNi phases.

Increasing the Ni content to 4% led to dense precipitation of Ni-containing phases, as noted in the backscattered image displayed in Figure 9a. Spot EDS spectra indicated possible precipitation of Al_9_FeNi (59 at%Al, 1.5at%Fe, 21.5 at%Ni, 18.2 at%Cu)—see the EDS spectrum shown in Figure 9b, corresponding to the black square in Figure 9a, and simultaneous precipitation of Al_3_Ni and Al_3_CuNi phases as shown in the EDS of Figure 9c, corresponding to the rectangular area outlined in red in Figure 9a.

### 3.3. HR-TEM Analysis

**Figure 10:**
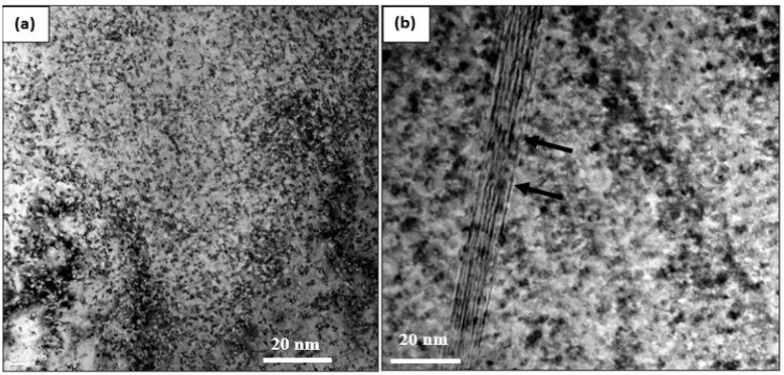

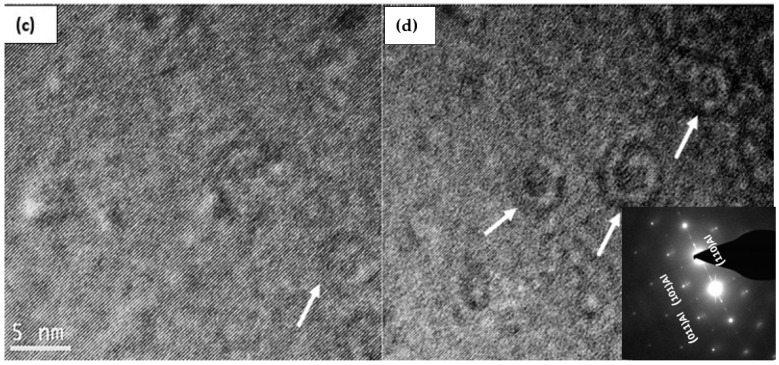
TEM micrographs of the B alloy sample aged for 8 h at 160 °C: (**a**,**b**) bright field electron images—note the presence of stacking fault lines in (**b**) caused by interruption in the order of stacking planes (marked by black arrows); (**c**) high resolution image showing coherency of the ultra-fine precipitates with the Al matrix; (**d**) high resolution image of (**a**)—inset micrograph is SAED-[111]Al zone axis.

In this section, samples heat-treated in the T6 and T7 conditions will be discussed, in addition to those exposed to a prior stabilizing treatment for 200 h at 240 °C. Figure 10a,b presents the precipitation in samples in the underaged condition (8 h at 160 °C) showing ultra-fine precipitates. These precipitates are still coherent with the aluminum matrix, as inferred from the high-resolution images displayed in Figure 10c,d, revealing contrast-like areas around the coherent precipitates (white arrows). It has been proposed that due to the high degree of coherency, extensive coherency–strain fields are developed, leading to an increase in the peak strength of the alloy. Thus, the formation of θ” precipitates may lead to distortion in the lattice structure in, and around, their vicinity. These distortions will impede the movement of dislocations during plastic deformation, resulting in hardening effects [14,22,77,78,79,80,81,82,83,84,85,86].

**Figure 11:**
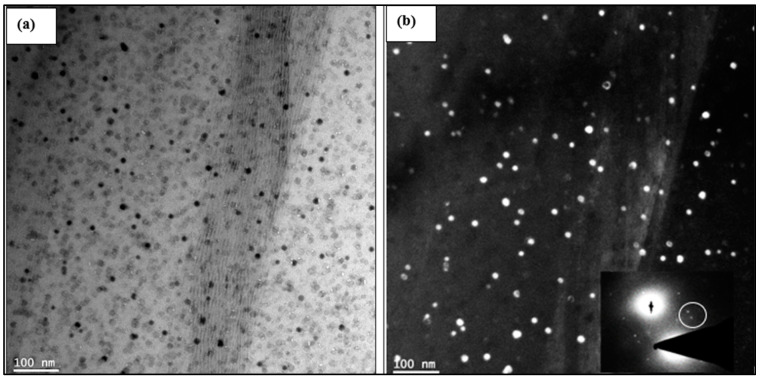
TEM images of the B alloy treated in the T6 tempered condition: (**a**) bright field, (**b**) corresponding dark field using double beam.

Figure 11 reveals further details of the precipitation in the same sample. Figure 11a shows a clearer image of the spherical precipitates (bright field), while Figure 11b shows the corresponding image in dark field mode. The associated selected area electron diffraction (SAED) pattern in the inset micrograph in Figure 11b reveals the presence of small spots (see white circled area) from which Figure 11b was generated, revealing the size and density of the precipitation that occurred during the applied aging treatment.

According to Starke [85] and Weatherly and Nicholson [86], fine θ″ particles would nucleate uniformly, with a strong coherency with the matrix. As a result of this process, strong strain fields similar to those shown in Figure 11a may be generated, resulting in significant enhancement of the alloy strength, as can be seen from Figure 2 and Figure 3. Such lattice structure distortion of the particles, as well as in the surrounding areas of the particles themselves, will restrict displacement of the dislocations, causing the observed hardening effects.

In addition, Starke [85] and Weatherly and Nicholson [86] reported that aging of aluminum alloys at elevated temperatures (overaging) would lead to changes in the microstructure that are independent of applied stress. These changes include the nucleation and growth of new phases as well as a reduction in the dislocation density due to the formation of sub-grains. Furthermore, coarsening of the precipitates may lead to the formation of soft precipitate-free zones along the grain boundaries, thereby disturbing the strength balance across the grains and causing premature crack formation. Thus, during this stage, the applied stresses are expected to enhance the precipitate coarsening rates.

**Figure 12:**
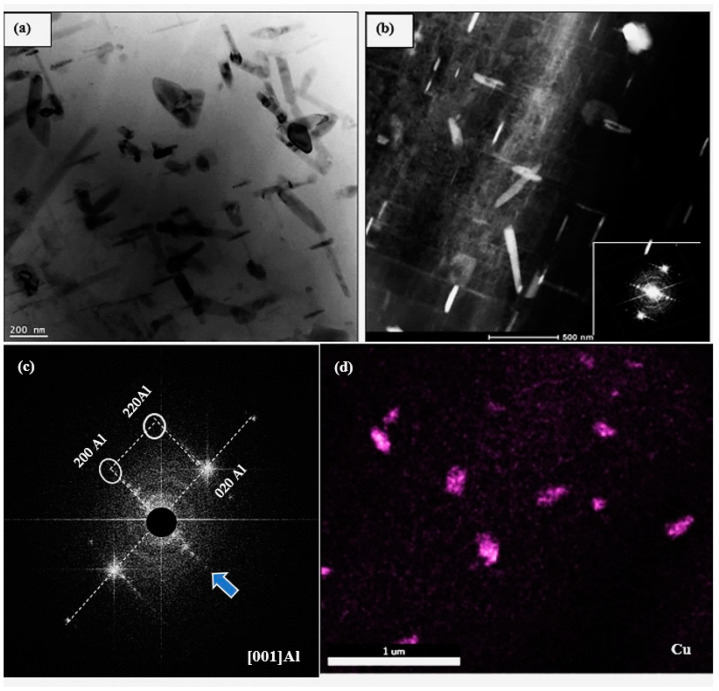
TEM micrographs of the B alloy aged at 240 °C for 50 h: (**a**) bright field, (**b**) dark field electron images, (**c**) the selected area electron diffraction (SAED) pattern obtained from the precipitates in (**a**)—note the presence of faint white spots (blue arrow). (**d**) X-ray image of Cu-rich particles in (**a**).

Figure 12a is a bright field micrograph of alloy B aged at 240 °C for 50 h, displaying precipitates in the form of short platelets coarser than those seen in Figure 11a. Figure 12b is a dark field image revealing the presence of various shapes of the precipitated phase particles in the microstructure. It should be mentioned here that these micrographs were prepared from samples following aging with no applied stress. The SAED pattern associated with Figure 12a is shown in Figure 12c. The zone axis is identified as [001]Al. Along the lines, several fine spots could not be identified with certainty. Some concentric rings can also be seen. Due to this difficulty, a colored map was produced, revealing that the large spots in Figure 12a could be identified as Cu-rich particles. The dark field image in Figure 12b was generated using fine spots in the white circles.

**Figure 13:**
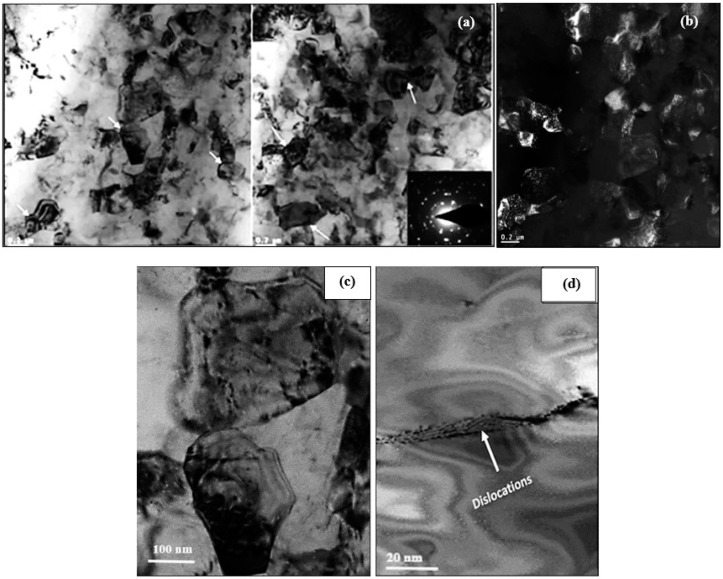

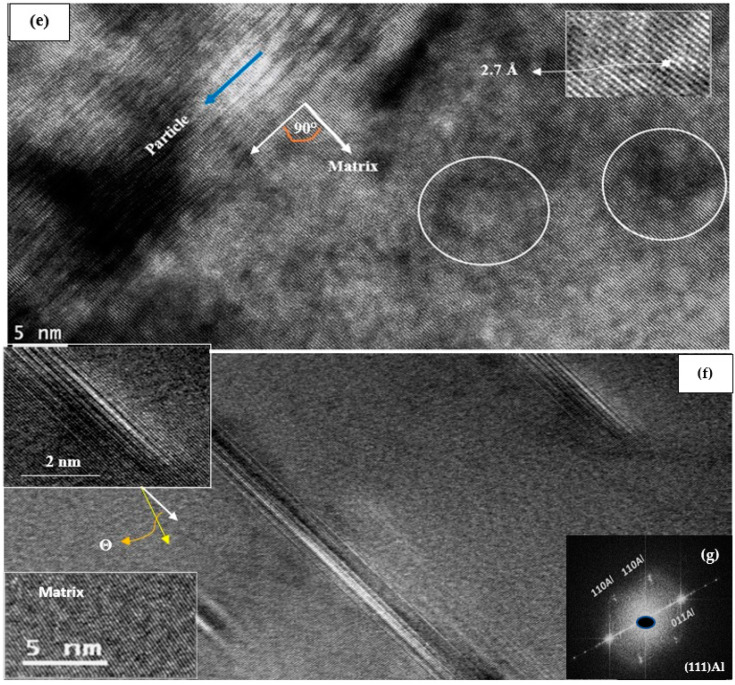
TEM images of the B alloy aged at 240 °C for 200 h: (**a**) composite bright field image, (**b**) corresponding dark field of (**a**); (**c**,**d**) bright field images showing an enlarged portion of two particles in (**a**); (**e**) high resolution electron image showing the transition from coherent particles (white circles) to incoherent ones (blue line); (**f**) high resolution electron image depicting the progressive increase in incoherent particles for samples tested at 240 °C—note all the three long precipitates are growing in the same direction; the white and yellow arrows-inset shows a gradual change in the interplanar orientation; (**g**) fast Fourier transition (FFT) pattern obtained for this image—extra points due to GPs/coherent precipitates.

Several coarsened particles over 200 nm can be seen in the bright-field images presented in composite form in Figure 13a. These particles reveal signs of particles colliding at their interfaces (white arrows) when the aging time at 240 °C was increased to 200 h—note the collision between the particles in the inset micrograph. The dark field image shown in Figure 13b indicates another feature: that is, not all particles have the same zone axis. This statement is supported by the SAED demonstrating several rings formed by a large number of spots. On enlarging Figure 13a, the image in Figure 13c shows interfaces between two colliding particles. Another point to be considered is the presence of dislocation arrays (arrowed) between two consecutive particles (low-angle boundary)—Figure 13d.

The high magnification image in Figure 13e reveals the crystallographic orientation-relationship between the precipitates and the surrounding matrix, where the two particle/matrix interfaces seem to possess crystallographic orientations that differ from the matrix, indicating the commencement of incoherency and the formation of Θ-phase particles. Based on the inset in Figure 13e, the inter-planar distance is about 2.7 Å. The tendency of transition of the precipitates from coherent to incoherency became more pronounced when the sample was tested at 240 °C, as shown in Figure 13f. Although the fast Fourier transition (FFT) pattern shown in the inset suggests strong coherency between the particle and the surrounding matrix, the observed deviation in the direction of the planes (Θ) indicates progressive incoherency.

**Figure 14:**
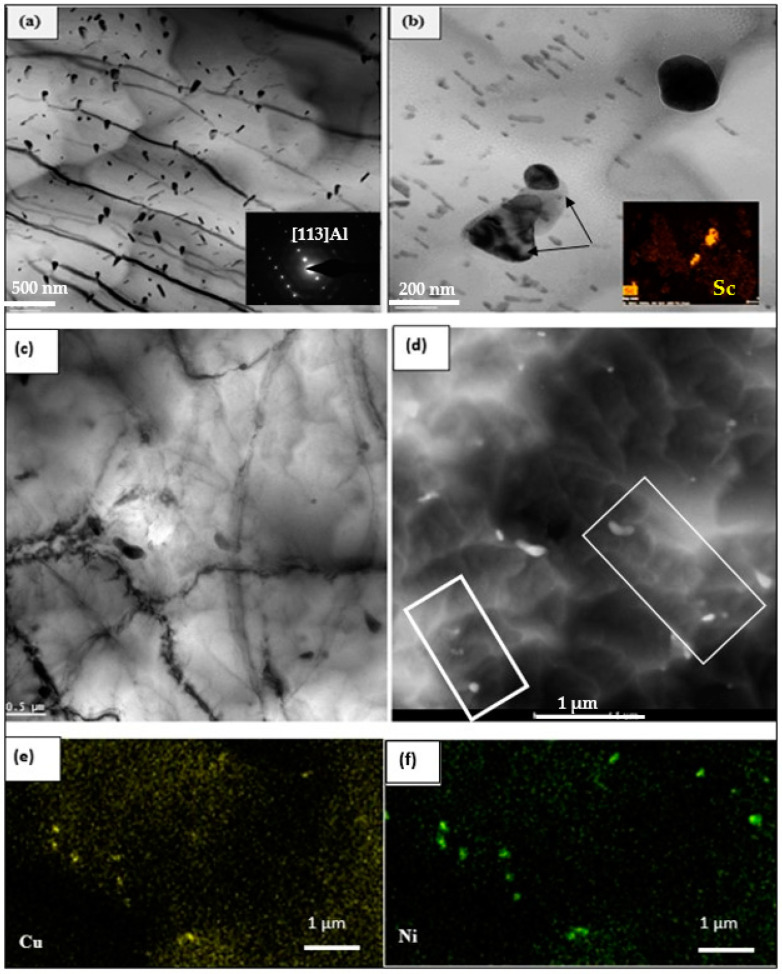

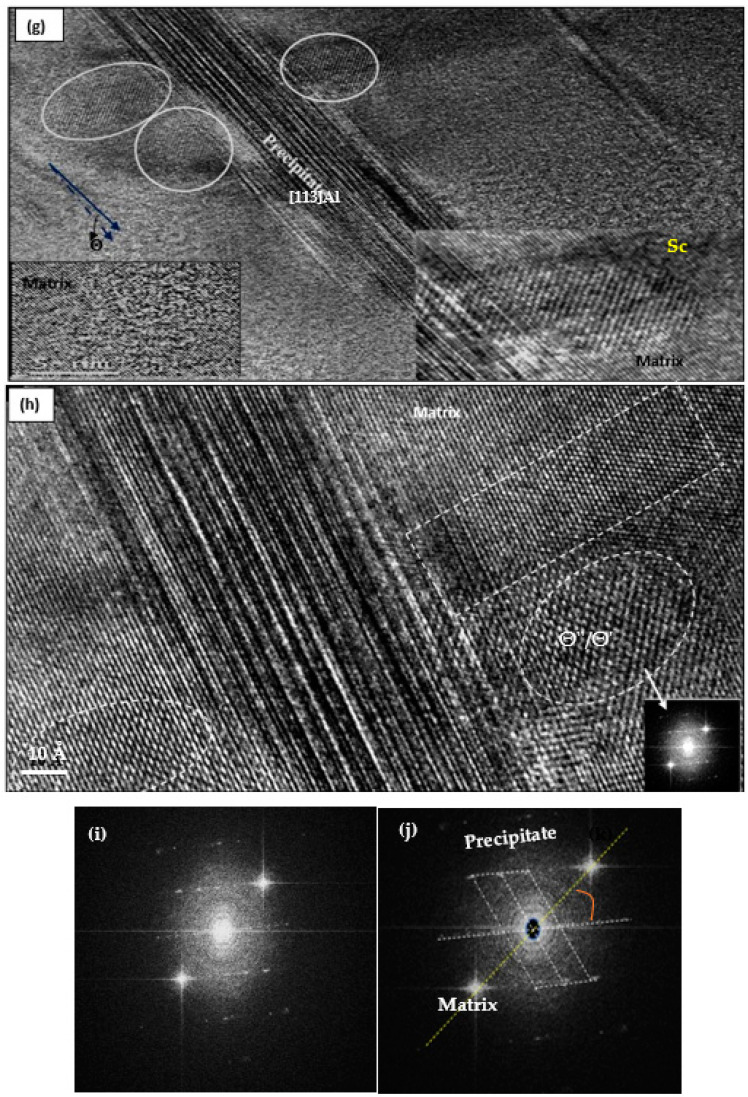
(**a**,**b**) TEM micrographs of the B2N alloy aged at 240 °C for 200 h bright field images—the black arrows in (**b**) point to Al_3_Sc particles; (**c**) high magnification electron image of (**a**); (**d**) dark field image of (**c**) note the presence of very fine particles; (**e**,**f**) X-ray maps showing the distribution of Cu and Ni in (**d**) rectangular frames in (**d**) indicate the areas from which the Cu–Ni particles were imaged for the X-ray mapping; (**g**) high-resolution electron image corresponding to (**a**); (**h**) a high magnification image taken from another area of the sample, confirming observations noted in (**g**), (**i**,**j**) fast Fourier transition (FFT) pattern corresponding to area marked Θ″/Θ′ in (**h**).

Figure 14a is a bright field image produced from the B2N alloy (the sample was aged at 240 °C for 200 h), depicting the distribution of Al_2_Cu particles formed by the remaining Cu in the matrix. As was shown in the SEM results presented in Section 3.2, a large amount of the Cu in the B2N alloy interacted with Ni, forming complex Al–Cu–Ni intermetallic particles, explaining the low volume fraction of the θ-Al_2_Cu particles in Figure 14a compared to that observed in Figure 12a. The bright field image shown in Figure 14b depicts the distribution of Sc- and Zr-rich particles, whereas Figure 14d is a dark field image obtained from Figure 14c. The X-ray maps in Figure 14e and Figure 14f show the distribution of Cu and Ni, respectively, in Figure 14c and highlighted by the tetragonal in Figure 14d. Figure 14g is a high-resolution electron image showing fusion/or nucleation of coherent particles (white circles) into/on an incoherent or semi-incoherent long particle, as depicted in the inset micrograph showing a difference between the direction of the planes of the particle and the surrounding matrix.

The small angle Θ between the solid and broken blue arrows indicates the *commencement* of incoherency of the large particle. The upper portion of Figure 14g was further enlarged, and is presented in Figure 14h, revealing fine GPII zones giving rise to streaking in the FFT (inset), in addition to most likely an early form of the Θ″ phase. In addition, the interplanar distance of the matrix is still around 2.7 Å. It should be mentioned here that Θ measured in Figure 14g is relatively smaller than that reported in Figure 13f due to delay in the onset of incoherency [85,86,87]. Figure 14i is the fast Fourier transition (FFT) pattern obtained for Θ″/Θ′ image in Figure 14h. At this stage, the precipitate forms a more symmetrical pattern (faint spots-white lines) with an angle with the yellow line (aluminum matrix) due to their coherency with the matrix. The precipitate has more closely packed diffraction patterns than the matrix, indicating that the precipitate has some planes with larger interplanar spacings than those of the aluminum matrix (bright spots). Idrac et al. [88] investigated the precipitation in Al–Cu system. Electron diffraction performed on all the model alloys detected only three phases. These phases are the α-Al, Θ-Al_2_Cu, and ȵ_2_-AlCu phases. The diffraction pattern presented in Figure 14i is close to some extent to [112]-Θ Al_2_Cu in refs. [86,87,88].

**Figure 15:**
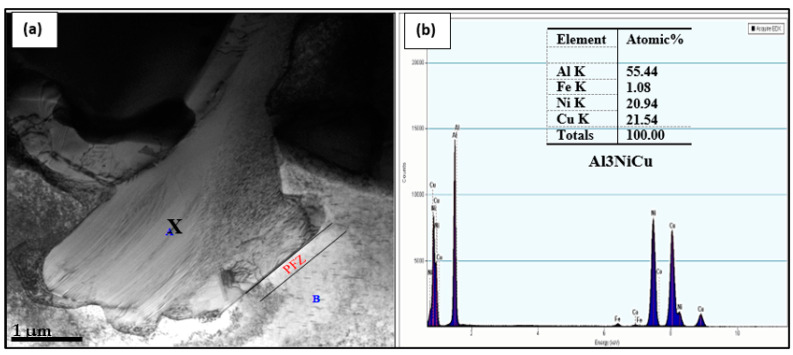
(**a**) Bright-field image of the B2N alloy sample aged for 200 h at 240 °C—revealing precipitation of a coarse Cu–Ni phase particle (marked A) in aluminum matrix (marked (B), (**b**) EDS spectrum corresponding to the point marked X in (**a**) showing strong peaks due to Al, Cu, and Ni.

**Figure 16:**
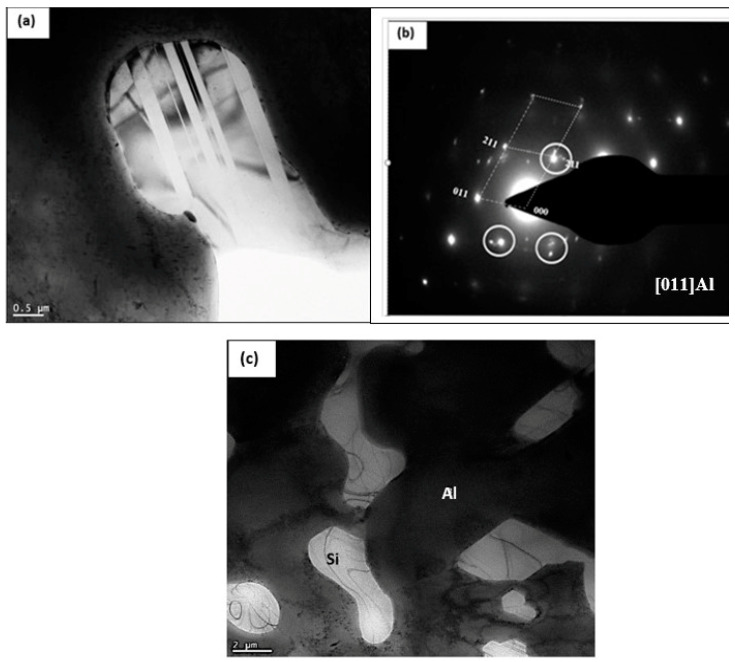
TEM micrograph of alloy the B2N alloy aged at 240 °C for 200 h: (**a**) bright field image-note the presence of twins within the Si particle, (**b**) selected area electron diffraction (SAED) pattern corresponding to (**a**) the white circles points to the presence of double zone axis caused by twinning, (**c**) general view of the Sr-modified eutectic Si particles.

The bright field micrograph presented in Figure 15a reveals the presence of a coarse Al–Cu–Ni particle, where precipitate-free zones (PFZs) emerged near the intermetallic particle areas. These zones are normally caused by coarse particles on the grain boundaries, leading to the depletion of Cu atoms in these zones [22,87,88,89,90,91,92]. Figure 15b is the EDS spectrum associated with the point marked X in Figure 15a, showing peaks due to Al, Cu, and Ni elements. According to Hofmeister [93] and Liu et al. [91], the observed twins in Figure 16a and confirmed by their corresponding selected area electron diffraction (SAED) pattern depicted in Figure 16b are fivefold twinned nanoparticles termed multiply-twinned particles (MTP) with composition of tetrahedra (Dh) decahedron and (Ic) icosahedron and crystallographic ordination of {1 1 1}/[1 1 2] type. Figure 16c displays the general distribution of Si and Al where the Si particles are having wavy boundaries instead of sharp ones observed in non-modified alloys.

## 4. Conclusions

Based on the findings presented in this study, the following conclusions may be drawn:

1. Aging of Al–Si–Cu–Mg alloys is characterized by the formation of multiple hardness peaks at the aging temperature with increasing aging time caused by simultaneous precipitation and coarsening of multiple phases.
2. Increasing the Ni content to 4 wt% improves the alloy resistance for softening during aging at 240 °C.
3. There are strong Cu–Ni, Zr–Ni, and Cu–Zr interactions between Cu, Ni, and Zr, causing a depletion in the matrix of Cu atoms needed for precipitation strengthening. This leads to a major reduction in the density of Al_2_Cu phase particles, and hence a lowering of the alloy strength.
4. The Ni-rich compounds are observed to precipitate on the surface of precipitated primary Zr-phase particles.
5. Aging the base 354 alloy at 240 °C for a long period of 200 h enhances the coarsening of the precipitated Al_2_Cu particles, leading to the commencement of incoherency.
6. In a 4 wt% Ni-containing alloy, a new phase was reported, namely Al_3_Ni_2_Cu_2_, representing almost 50% (about 50 at%) of the compound composition.
7. Silicon particles were characterized by the presence of a large number of twins.
8. In the precipitation of primary Cu–Ni-rich particles, the particles are surrounded by precipitate-free zones due to depletion of Cu atoms in their vicinity.

## Figures and Tables

**Table 1:** Chemical analysis (wt%) of the alloys used in the present study.

Elements (wt%) *^,^**										
Alloy Code	Si	Cu	Mg	Fe	Ti	Zr	Ni	Ag	Sr	Al
B	8.5	2.0	0.59	0.15	0.15	0.35	0.1	0.68	--	Bal
BS	8.6	1.8	0.48	0.15	0.18	0.23	0.1	0.67	0.017	Bal
B2N	8.5	1.8	0.58	0.13	0.18	0.23	2.0	0.68	0.015	Bal
B4N	8.7	1.9	0.68	0.2	0.20	0.30	4.0	0.72	0.017	Bal

* All alloys contain about 0.06%Sc to enhance grain refining; ** Major elemental differences in the alloys.

**Table 2:** Details of the applied heat treatments.

(**a**)		
**Treatment #**	**Description**	
1	AC	As cast (AC) T5
2	AC + 8 h/240 °C	
3	AC + 110 h/240 °C	
4	AC + 200 h/240 °C	
5	SHT + 8 h/240 °C	Solution heat treatment (SHT) 495 °C/5 h/quenching in warm water (60 °C) T7
(**b**)		
**Treatment #**	**Description**	
1	AC	As cast (AC) T5
2	AC + 8 h/240 °C	
3	AC + 110 h/240 °C	
4	AC + 200 h/240 °C	
5	SHT + 8 h/240 °C	Solution heat treatment (SHT) 495 °C/5 h/quenching in warm water (60 °C) T7
6	SHT + 50 h/240 °C	
7	SHT + 110 h/240 °C	
8	SHT + 200 h/240 °C	

**Table 3:** Main reactions taking place during solidification of alloys B and B2N [57].

Reaction #	Suggested Temperature Range (°C)	Suggested Precipitated Phase
1	600–597	Formation of *α*-aluminum dendritic network
2	560–558	Precipitation of Al-Si eutectic Precipitation of post-eutectic *β*-Al_5_FeSi phase
3	555–556	Precipitation of Al_9_FeNi phase
4	540–538	Precipitation of Mg_2_Si phase
5	525–523	Transforming of *β*-phase into *π*-Al_8_Mg_3_FeSi_6_ phase
6	523–520	Precipitation of Al_3_CuNi phase
7	500–496	Formation of eutectic Al-Al_2_Cu phase
8	485–489	Precipitation of *Q*-Al_5_Mg_8_Cu_2_Si_6_ phase

**Table 4:** WDS analysis of phases reported in alloy B.

Alloy Code	Element	at%	Calculated Formula	Suggested Formula
B	Al	51.51	Al_11.6_Fe_6_Mg_4.02_Si_4.7_	π-Al_8_Mg_3_FeSi_6_
	Fe	4.85		
	Mg	19.53		
	Si	23.12		
	Total	99.01		
	Al	68.11	Al_2.25_Cu	Θ-Al_2_Cu
	Cu	30.21		
	Total	98.31		
	Mg	11.14	Al_4.08_Cu_2_Mg_9.2_Si_7_	Q-Al_5_Mg_8_Cu_2_Si_6_
	Al	47.41		
	Si	8.70		
	Cu	33.74		
	Total	100.00		
	Mg	24.82	Mg_2.1_Si	Mg_2_Si
	Al	45.23		
	Si	21.98		
	Cu	7.97		
	Total	100.1		

**Table 5:** WDS analysis of phases observed in alloy B2N.

Alloy Code	Element	wt%	at%	Calculated Formula	Suggested Formula
B2N	Al	37.67	58.41	Al_5.88_Si_1.34_Ti_0.25_Zr_2.14_	(Al,Si)_3_(Ti,Zr)
	Si	11.94	16.29		
	Ti	3.79	3.31		
	Zr	47.14	21.62		
	Total	99.54	99.63		
	Al	66.69	80.44	Al_8.94_Fe_0.25_Ni_1.7_	Al_9_(FeNi)
	Fe	3.86	2.25		
	Ni	27.62	15.31		
	Total	99.85	98.86		
	Al	40.77	61.03	Al_3.30_Ni_1.13_Cu	Al_3_CuNi
	Ni	29.77	20.48		
	Cu	28.44	18.07		
	Total	98.98	99.58		

**Table 7:** Volume percentage (%) of undissolved phases in as-cast and T4-tempered alloys.

Condition	Volume Fraction (%)		
Alloy	B	B2N	B4N
As cast	2.5 ± 0.4	12.2 ± 0.8	15.3 ± 0.8
T4	1.2 ± 0.3	9.5 ± 0.7	11.5 ± 0.6

## Data Availability

The original contributions presented in the study are included in the article. Further inquiries may be directed to the corresponding author.
